# Diffuse myocardial fibrosis using native T1 mapping in children with repaired Tetralogy of Fallot: correlation with surgical factors and exercise capacity

**DOI:** 10.1186/1532-429X-18-S1-O27

**Published:** 2016-01-27

**Authors:** Deane Yim, Eugenie Riesenkampff, Shi-Joon Yoo, Mike Seed, Lars Grosse-Wortmann

**Affiliations:** grid.42327.300000000404739646Paediatric Cardiology, Hospital for Sick Children, Toronto, ON Canada

## Background

Regional myocardial scarring in adults following Tetralogy of Fallot (TOF) repair is associated with adverse clinical outcomes. This is the largest study to date assessing native T1 times as a marker for diffuse myocardial fibrosis in children after TOF repair. The aim of the study was to describe native T1 times patients post-TOF repair and to evaluate its relationship to clinical status and surgical factors.

## Methods

Mid-ventricular native T1 times using a modified look-locker inversion recovery (MOLLI) sequence were obtained in 80 consecutive children post-TOF repair, aged 13.1 ± 2.8 years, during routine cardiac magnetic resonance imaging (CMR). CMR results were compared to 28 controls and associations between native T1 times and echocardiographic, surgical and exercise factors were studied.

## Results

Patients and controls were similar in age and gender distribution. Expectedly, TOF patients had significantly increased indexed right ventricular end-diastolic and end-systolic volumes and, as has been reported, significantly reduced right and left ventricular ejection fractions on CMR compared to controls. Increased right mid-ventricular native T1 times were found in cases compared to controls (1041 ± 78 vs 959 ± 33 ms, p < 0.001). There were no significant differences in the left mid-ventricular native T1 times between patients and controls. Primary repair bypass and cross-clamp times correlated with native T1 times of the left ventricular myocardium (r = 0.30 for bypass and 0.32 for cross clamp times), interventricular septum (r = 0.34 and 0.32 respectively), left ventricular free wall (r = 0.31 and 0.47 respectively) and right ventricle (r = 0.29 for cross clamp time). Weak negative correlations were observed between right ventricular native T1 times and right ventricular outflow tract gradient by echocardiography (r= -0.28), indexed left end-diastolic volume and stroke volume on CMR (r= -0.26 and -0.28 respectively). There was no correlation between native T1 times and the age or weight at primary repair, type of repair, QRS duration or peak predicted oxygen consumption or workload.

## Conclusions

Children following TOF repair have evidence of diffuse right ventricular myocardial fibrosis as demonstrated by higher mid-ventricular native T1 times compared to controls. Global left ventricular native T1 times are associated with longer aortic bypass and cross-clamp times at the time of primary repair. Clinical correlations with exercise capacity were not observed.

**Figure 1:**
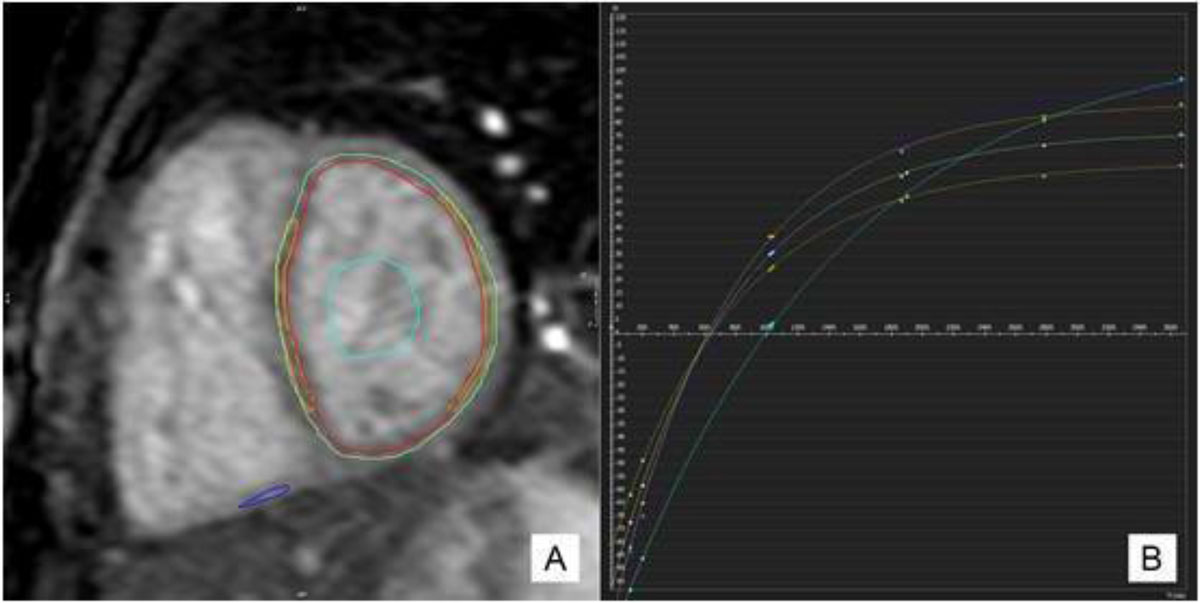
**(A) T1 mapping contours and (B) derived exponential time curves plotting the native T1 times on the X-axis and signal intensity (SI) on the Y-axis in a patient post-TOF repair**. The curve is 1 of 8 mid-ventricular images acquired at varying inversion times that collectively make up the MOLLI sequence. Regions of interest are drawn within the left ventricular myocardium (red and green), interventriculal septum (orange), blood pool (light blue) and the native T1 times are calculated using a curve-fitting algorithm.

